# Pneumatosis intestinalis with free air in the abdominal cavity caused by nintedanib

**DOI:** 10.1002/rcr2.1085

**Published:** 2023-01-30

**Authors:** Naho Sakamoto, Motoyasu Kato, Toshihiko Oni, Yuriko Terayama, Shun Nakazawa, Shouichi Okamoto, Jun Ito, Kazuhisa Takahashi

**Affiliations:** ^1^ Department of Respiratory Medicine Juntendo University Graduate School of Medicine Tokyo Japan; ^2^ Department of Respiratory Medicine Saiseikai Ibaraki Hospital Ibaraki Japan

**Keywords:** adverse effects, idiopathic pulmonary fibrosis, nintedanib, pneumatosis intestinalis

## Abstract

This is the first case confirmed the association between PI development and nintedanib by the reproducibility of PI development. In patients taking a combination treatment with corticosteroid and nintedanib, clinicians should be careful regarding the development of PI although the patient improved only after discontinuation of nintedanib treatment.

## Graphical abstract

This is the first case confirming the association between pneumatosis intestinalis (PI) development and nintedanib by the reproducibility of PI development. In patients taking a combination treatment with corticosteroid and nintedanib, clinicians should be careful regarding the development of PI although the patient improved only after discontinuation of nintedanib treatment.

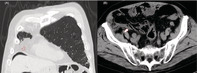

## CLINICAL IMAGE

**FIGURE 1:**
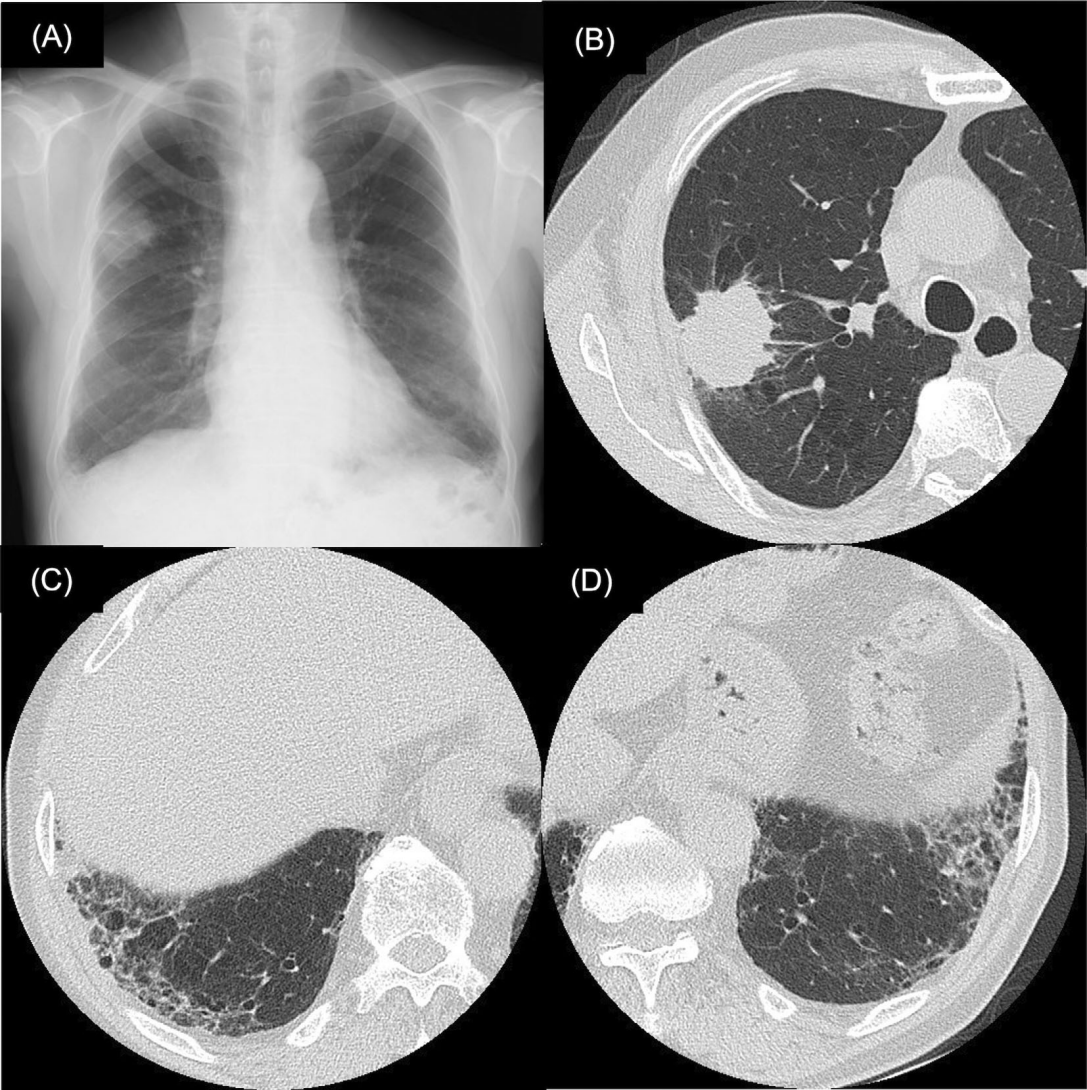
(A) Chest X‐ray findings at the time of referral to our hospital. High‐resolution computed tomography in the upper lung (B) and lower lung (C, D)

**FIGURE 2:**
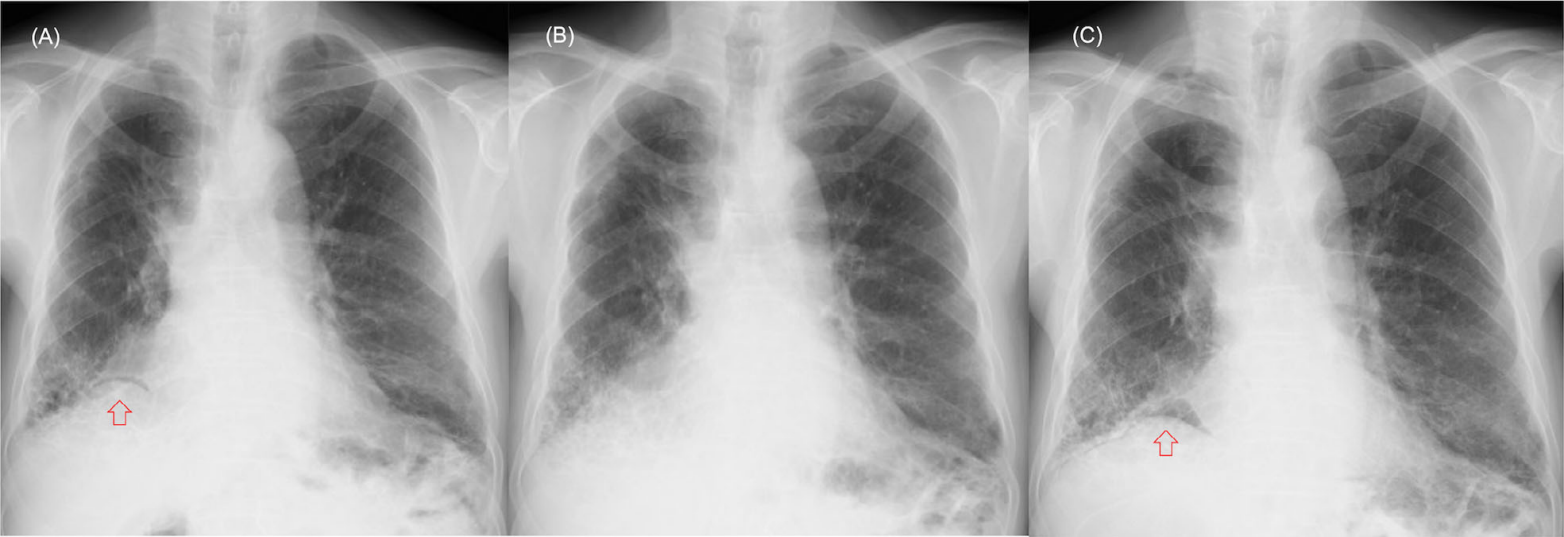
Chest X‐ray findings at the development of pneumatosis intestinalis (PI) (A), at the improvement of PI (B), and at the relapse of PI (C). The arrows show free air in (A) and (C)

**FIGURE 3:**
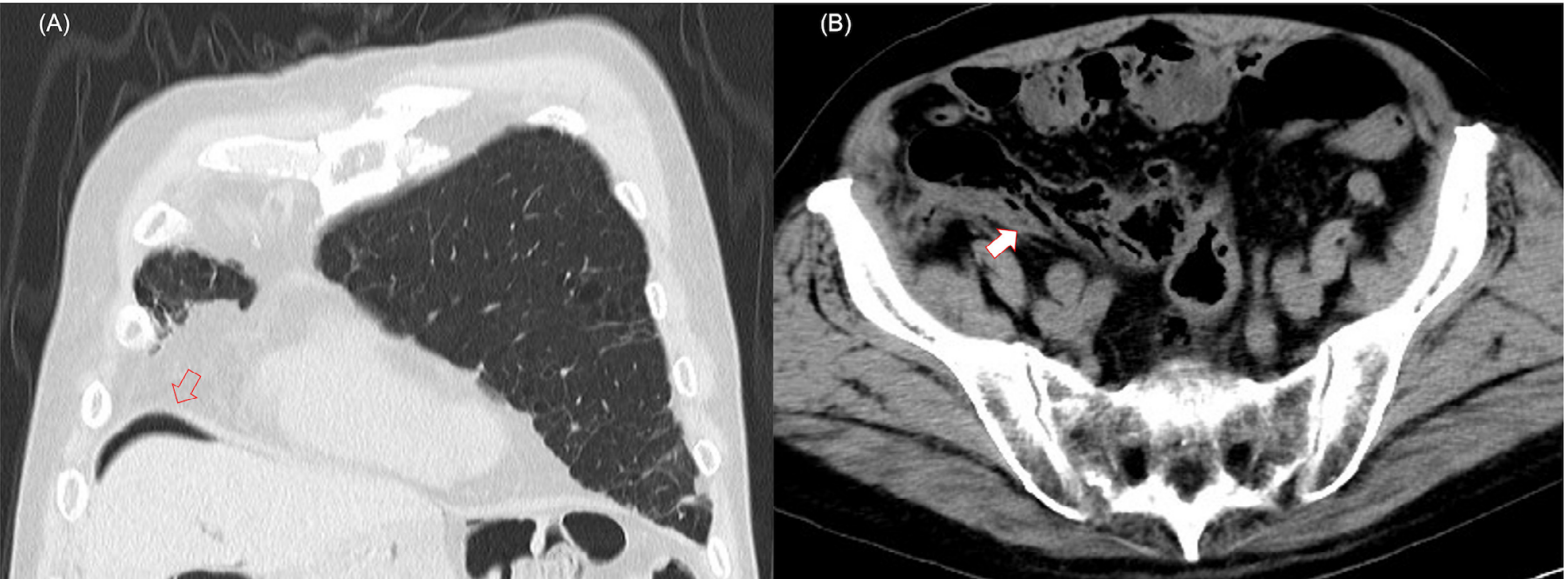
(A) Axial computed tomography lung window images at the development of free air in pneumatosis intestinalis (PI). The arrows show free air just below the diaphragm. (B) Transverse view of abdominal computed tomography: arrow shows PI in the intestinal wall

An 83‐year‐old man who was diagnosed with both idiopathic pulmonary fibrosis (IPF) and non‐small cell lung cancer underwent lobectomy and developed acute exacerbation of IPF after this surgery (Figure 1); thus, he received corticosteroids. Three years after the corticosteroid initiation, nintedanib was initiated. Twenty‐two months after the nintedanib initiation, his radiological examination accidentally revealed free air and pneumatosis intestinalis (PI) without any abdominal abnormal symptoms including diarrhoea, and physical findings (Figures 2A and 3). Based on the absence of abdominal symptoms and the changes in laboratory data, we and the surgeon concluded the abnormal X‐ray findings were not caused by intestinal perforation. Two weeks after discontinuing nintedanib, the free air disappeared (Figure 2B). We re‐initiated nintedanib, however; the X‐ray re‐showed an appearance of the free air (Figure 2C). Therefore, we considered nintedanib administration was significantly associated with PI development. Only one case was ever reported to develop PI during nintedanib treatment.^1^ The mechanism associated with PI development and nintedanib is suggested to be tissue fragility caused by the inhibition of angiogenesis as well as the association with pneumothorax in previous reports. In this case, the tissue might be fragile due to long‐term steroid therapy. Additionally, the inhibition of angiogenesis by nintedanib may predispose microscopic ischemic changes in the intestinal mucosa, resulting in PI. Using registry data,^2^ we recommend nintedanib treatment with careful observation of pneumothorax and PI.

## AUTHOR CONTRIBUTIONS

Naho Sakamoto, Motoyasu Kato, Toshihiko Oni, Yuriko Terayama, Shun Nakazawa, Shouichi Okamoto, and Jun Ito were the attending doctors who treated the patient. Motoyasu Kato was the outpatient doctor. Naho Sakamoto, Motoyasu Kato, Jun Ito, and Kazuhisa Takahashi managed all treatment for the patient. Naho Sakamoto and Motoyasu Kato drafted the manuscript. Naho Sakamoto submitted the final manuscript. All authors read and approved the final manuscript

## CONFLICT OF INTEREST

Motoyasu Kato received research funding, speaker horneria, and consultation fee from Boehringer Ingelheim. Kazuhisa Takahashi received research funding from Boehringer Ingelheim.

## ETHICS STATEMENT

The authors declare that appropriate written informed consent was obtained for the publication of this manuscript and accompanying images.

## Data Availability

Data sharing not applicable to this article as no datasets were generated or analysed during the current study.

## References

[1] Sidney SB . Pneumatosis intestinalis associated with the tyrosine kinase inhibitor Nintedanib. Lung. 2018;196:373–5. 10.1007/s00408-018-0118-6 29721603

[2] Richeldi L , du Bois RM , Raghu G , Azuma A et al. Efficacy and safety of nintedanib in idiopathic pulmonary fibrosis. N Engl J Med. 2014;370:2071–82. 10.1056/NEJMoa1402584 24836310

